# Transvaginal resection of a gastrointestinal stromal tumor of the rectum: a case report

**DOI:** 10.1186/s40792-024-01949-z

**Published:** 2024-06-18

**Authors:** Sanshiro Hatai, Shuntaro Nagai, Taiki Yoshida, Masaru Matsuoka, Tomohiko Shinkawa, Yasuhiro Oyama, Yoshitaka Tanabe, Daichi Kitahara, Sadafumi Tamiya, Satoshi Amada, Kazuyoshi Nishihara, Toru Nakano

**Affiliations:** 1 Department of Surgery, Kitakyushu Municipal Medical Center, 2-1-1 Basyaku, Kokurakita-Ku, Kitakyushu, Fukuoka 802-0077 Japan; 2 Department of Pathology, Kitakyushu Municipal Medical Center, Fukuoka, Japan; 3 Department of Obstetrics and Gynecology, Kitakyushu Municipal Medical Center, Fukuoka, Japan

**Keywords:** Transvaginal approach, Gastrointestinal stromal tumor, Minimally invasive surgery

## Abstract

**Background:**

The most common curative treatment for gastrointestinal stromal tumors (GISTs) is local excision. For rectal GISTs, however, local excision is difficult because of the anatomical features of the rectum. The optimal surgical approach is still under debate, and less invasive methods are desired. We herein report a case of transvaginal resection of a rectal GIST in a young woman.

**Case presentation:**

A 21-year-old woman was diagnosed with a resectable GIST in the anterior rectal wall and underwent transvaginal tumor resection. The posterior vaginal wall was incised, revealing the tumor fully covered by the rectal mucosa. The rectal adventitia and muscular layer were incised, and the tumor was resected en bloc without rupture. The postoperative course was favorable, and the patient was discharged on postoperative day 12. No findings consistent with recurrence were present 6 months postoperatively.

**Conclusion:**

Transvaginal tumor resection is a treatment option as a minimally invasive procedure for GISTs in the anterior rectal wall in female patients.

## Background

Gastrointestinal stromal tumors (GISTs) are mesenchymal tumors occurring most commonly in the stomach and small intestine. The rectum is the third most common site [1–3]. Many reports suggest that local excision like wedge resection with functional preservation is sufficient for a localized GIST in the upper gastrointestinal tract or colon [4–6]. However, local excision of a rectal GIST is difficult because of the anatomical features of the rectum, and the optimal surgical procedure is still under discussion. Although low anterior or abdominoperineal resection may be performed, such surgeries can lead to serious complications and may require ileostomy or colostomy, which increases the burden on patients. Therefore, a less invasive procedure is desirable. We herein report transvaginal resection of a rectal GIST with preservation of the rectal mucosa in a young woman.

## Case presentation

**Fig. 1:**
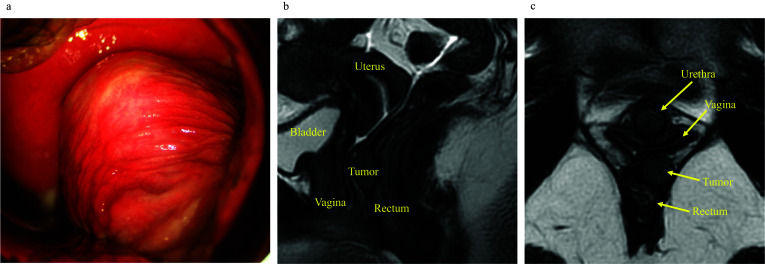
Preoperative findings. **a** The tumor observed transvaginally. **b** Preoperative magnetic resonance imaging findings (T2, sagittal). **c** Preoperative magnetic resonance imaging findings (T2, axial)

A 21-year-old woman was referred to our hospital for evaluation and treatment of a vaginal mass. On vaginal examination, the mass was recognized as an elevated lesion in the posterior wall of the vagina (Fig. 1a). A digital rectal examination revealed the mass in the anterior wall of the rectum approximately 4 cm from the anal verge. Colonoscopy showed a 15-mm submucosal tumor in the anterior wall of the rectum, and endoscopic ultrasound-guided fine needle aspiration cytology from the tumor revealed a GIST. There were no findings consistent with ulceration or mucosal invasion. On magnetic resonance imaging, the tumor was located in the rectum below the peritoneal reflection, and the posterior wall of the vagina was compressed by the tumor (Fig. 1b and c). The continuity of the vaginal wall was preserved, and there were no findings indicative of vaginal invasion by the tumor. A computed tomography scan showed neither enlarged lymph nodes nor distant metastasis.

We diagnosed a localized low rectal GIST and planned to perform surgery. Low anterior resection was considered excessive because of the highly invasive nature of this procedure, including complications such as infertility, urinary incontinence, and other urogenital dysfunctions, as well as the possibility of requiring ileostomy or colostomy. Transanal tumor resection and transanal endoscopic microsurgery were also options; however, transvaginal tumor resection was considered more advantageous for securing the operative field because of the tumor location. Therefore, we selected transvaginal tumor resection.

**Fig. 2:**
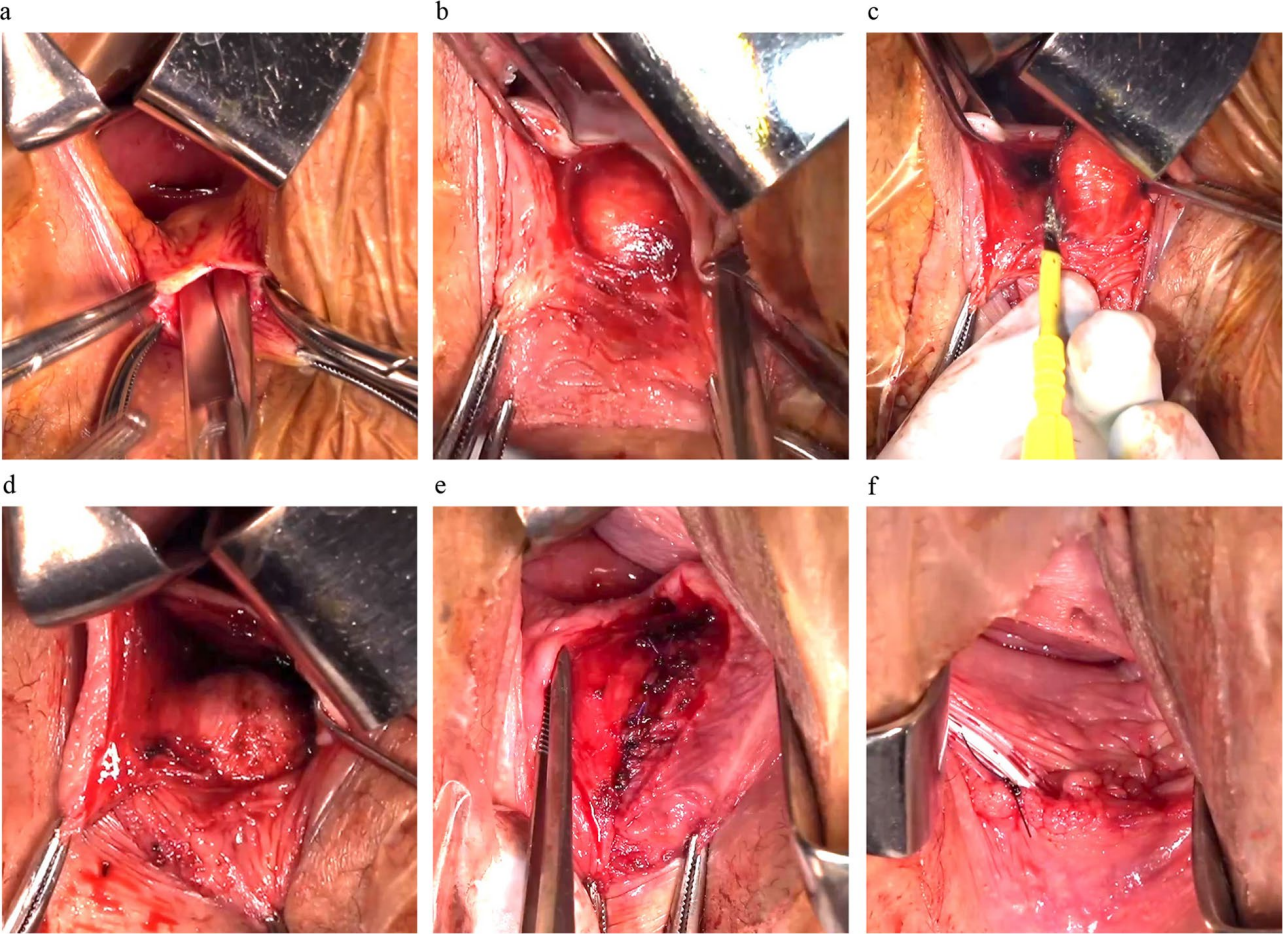
Operative findings. **a** A small transverse incision was made in the posterior wall of the vagina at the level of the hymenal ring. The posterior vaginal wall was then separated from the rectovaginal septum, and the posterior vaginal wall was vertically incised toward the cervix. **b** The tumor was completely exposed, covered by the rectal adventitia. **c** The tumor was easily mobilized with preservation of the tumor capsule. The dissection was aided by pushing the tumor anteriorly from the rectum. Detachment was performed multidirectionally. **d** Findings after tumor resection. There was no defect in the rectal mucosa. **e** Findings after suturing of the rectal adventitia and muscular layer. **f** Findings after suturing of the vaginal wall and placement of a drain

**Fig.3:**
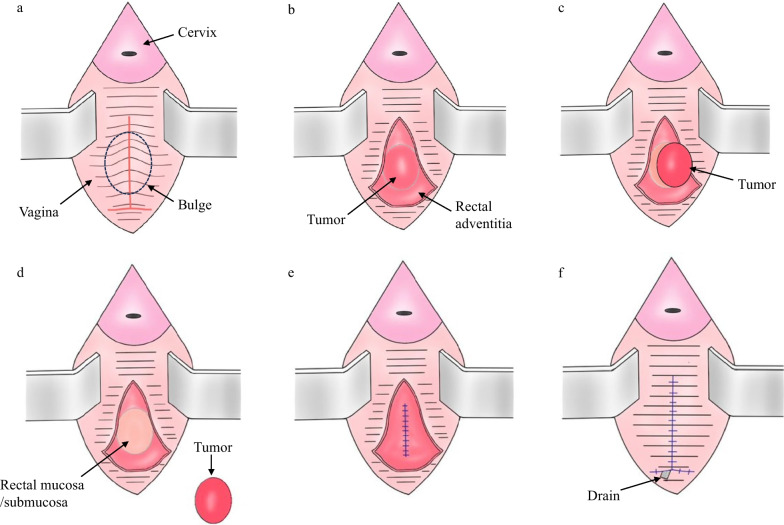
Surgical Procedure Schema. **a** An incision was made on the posterior wall of the vagina, confirming vaginal bulge. The posterior vaginal wall was separated from the rectovaginal septum. **b** The rectum adventitia was exposed, and the tumor was identified under the layer. **c** The tumor was mobilized with preservation of the tumor capsule. **d** The tumor was resected without any defect of the rectal mucosa. **e** The rectal adventitia and muscular layer was sutured. **f** The vaginal wall was sutured and a drain was placed

The surgery was performed with the patient under general anesthesia and in the lithotomy position. Vaginal examination revealed the rectal tumor as a mass near the vaginal opening. A small transverse incision was made at the posterior wall of the vagina at the level of the hymenal ring. The posterior vaginal wall was then separated from the rectovaginal septum, and the posterior vagina wall was incised vertically toward the cervix over the complete length of the tumor (Fig. 2a). With this procedure, the tumor was clearly recognized as a bulge covered only by the rectal adventitia and muscular layer (Fig. 2b). An incision was made in the rectal adventitia and muscular layer near the cranial edge of the mass. The tumor was easily mobilized while keeping the tumor capsule intact (Fig. 2c). The dissection was aided by pushing the tumor anteriorly from the rectum. Detachment was performed multidirectionally, and the tumor was resected en bloc without rupture. The procedure was completed with no defect of the rectal mucosa (Fig. 2d). The rectal adventitia and muscular layer were sutured using a single ligatured suture with 4-0 absorbable thread to cover the exposed mucosa (Fig. 2e). The surgical wound was cleansed with 100 mL of saline. The vaginal wall was then sutured using a single ligatured suture with 4-0 absorbable thread, and a drain was placed between the vaginal wall and the rectum from the most inferior portion of the wound (Fig. 2f). Finally, a piece of gauze was inserted into the vagina for hemostasis. The operation time was 50 min and the blood loss was 5 mL. The surgical procedure is shown in the schema (Fig. 3a–f).

**Fig. 4:**
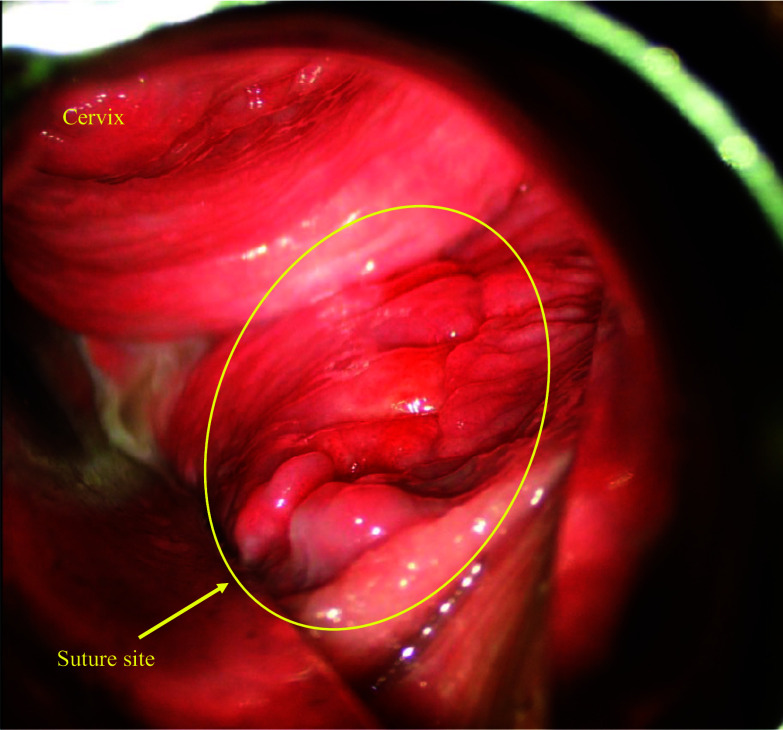
Postoperative findings. The wound site on postoperative day 17

The postoperative course was unremarkable. The day after the operation, the intravaginal gauze was removed after confirming hemostasis. The drain was removed on postoperative day 3. The patient resumed eating on postoperative day 7 and was discharged from the hospital on postoperative day 12. On postoperative day 17, the wound site was checked on an outpatient basis, confirming the absence of bleeding, wound dehiscence, infection, or a rectovaginal fistula (Fig. 4).

**Fig. 5:**
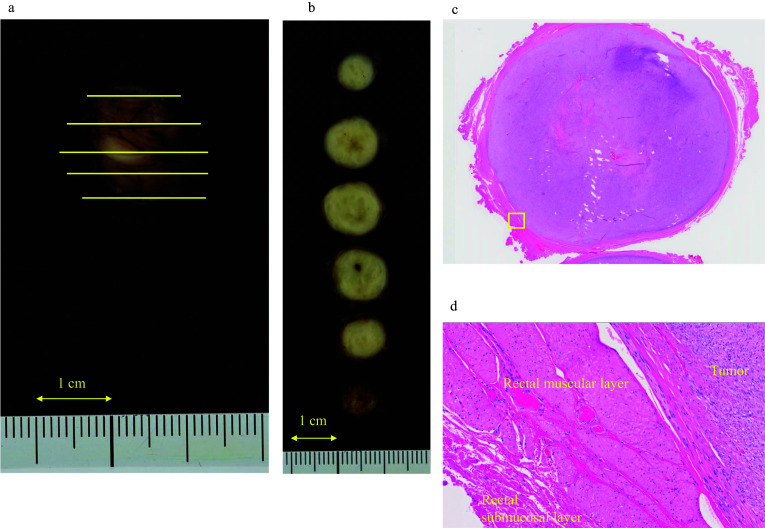
Histopathological findings. **a** Gross tumor findings. **b** Findings on the cut surface of the tumor. The area circled by the blue line is the tumor. **c** Findings observed with a loupe after hematoxylin–eosin staining. The tumor capsule was circumferentially preserved. **d** An enlarged image of the area circled by the yellow square of **c**. Findings of the surgical margin on the rectal mucosal side (hematoxylin–eosin staining, × 200)

Pathological examination showed a GIST measuring 18 mm in diameter and consisting of spindle-shaped cells. Tumor rupture was not seen, and the surgical margin was clear of tumor cells (Fig. 5a–d). Immunohistochemically, the tumor cells were positive for c-kit, DOG1, CD34, and smooth muscle actin and negative for S-100 protein and desmin. The tumor showed an average mitotic count of 1 per 50 high-power fields. Therefore, the tumor was considered very low risk according to the modified Fletcher classification [7, 8] and no risk according to the Miettinen classification [9]. The patient was followed up without chemotherapy, and no signs of recurrence were found 6 months after the surgery.

## Discussion

In this report, we have described our experience of transvaginal tumor resection for a rectal GIST. If possible, the mucous membrane should be preserved to avoid contaminating the surgical field with the intestinal contents as well as to reduce the risk of postoperative complications. To date, eight cases of transvaginal resection of a rectal GIST have been reported, and the rectal mucosa was preserved in only three of these cases.

GISTs are stromal tumors that originate from the interstitial cells of Cajal. The clinical practice guidelines on GIST from the European Society for Medical Oncology indicate that the standard treatment of localized GISTs is complete surgical excision of the lesion [10]. Many reports suggest that local excision with no dissection of clinically negative lymph nodes is sufficient for localized GISTs because GISTs rarely infiltrate the surrounding tissues or metastasize to the lymph nodes [4–6]. However, local excision of rectal GISTs is difficult because of the anatomical features of the rectum, and the optimal surgical procedure is still under debate. Depending on the size and location of the tumor, highly invasive surgery such as low anterior or abdominoperineal resection may be performed. Such procedures can lead to serious complications and may require ileostomy or colostomy, which increases the burden on patients. Therefore, a less invasive procedure is desirable. The usefulness of transanal resection [11] or transanal minimally invasive surgery [12, 13] has been reported; however, depending on the size and location of the tumor, it may be difficult to perform these procedures. If the tumor is located on the anterior rectal wall in a female patient, as in the present report, the transvaginal approach may be most appropriate. In transvaginal tumor resection, it is easy to secure the operative field of view because the vaginal wall is a flexible and elastic tissue. In addition, the tumor can be palpated through the rectum intraoperatively, making it easier to recognize the outer edge of the tumor. If it is difficult to secure the visual field, the use of retraction instrument may be also considered. In this case, because the field of view was sufficiently secured, we did not use it.

Transvaginal resection of various rectal tumors has been reported previously. Vorobyov et al. [14] reported a case of transvaginal resection of a rectal leiomyoma. Fu et al. [15] reported transvaginal resection of T1 and T2 rectal cancer in 18 patients and noted a lower complication rate with transvaginal than transanal rectal cancer resection. In our search of PubMed using the key words “transvaginal” and “GIST”, we found eight cases of transvaginal resection of a rectal GIST [16–22] (Table 1). In all cases, the surgery was performed safely and the postoperative course was favorable. The surgical margin was positive in one case in which a 10-cm GIST was resected [22], but the surgical margin was negative in the other cases. A larger tumor size makes surgery more difficult and increases the risk of positive margins and complications; therefore, preoperative chemotherapy should be considered to reduce the size of the tumor. Although the transanal approach is indicated for tumors up to 5 cm in diameter [17], there are currently no reports that clearly indicate the indications for transvaginal tumor resection by tumor size. Transvaginal tumor resection can safely remove even slightly larger tumors than is possible with transanal tumor resection because of greater vaginal dilation, a wider field of view, and a larger opening for specimen collection. Accumulation of additional cases is needed to determine at what tumor size transvaginal tumor resection can be safely performed and at what size preoperative chemotherapy is recommended.

**Table 1:** Previously reported cases of transvaginal resection of a rectal gastrointestinal stromal tumor

Author, year	Age	AV (cm)	Size (mm)	Operation time (min)	Blood loss (ml)	Preservation of rectal mucosa	Surgical margin	Discharge (POD)	Recurrence, follow-up month
Hellan, 2006 [16]	NA	3	80	120	NA	Yes	Negative	3	No recurrence, 6
Hara, 2011 [17]	45	6	48	110	24	No	Negative	7	NA
Shizhuo, 2019 [18]	62	3	50	NA	NA	Yes	Negative	NA	No recurrence, 24
Shizhuo, 2019 [18]	69	3	40	NA	NA	No	Negative	5	No recurrence, 12
Ferria, 2021 [19]	81	2.9	51	NA	NA	No	Negative	6	NA
Marino, 2022 [20]	69	4	28	NA	NA	Yes	Negative	NA	No recurrence, 6
Penninga, 2023 [21]	70	NA	40	NA	NA	No	Negative	2	No recurrence, 12
Feng, 2023 [22]	49	NA	100	NA	NA	No	Positive	NA	NA
Present study case	21	4	15	50	5	Yes	Negative	12	No recurrence, 6

*AV* anal verge, *POD* postoperative day, *NA* not available

Among the eight above-mentioned cases of transvaginal resection of a rectal GIST, the rectal mucosa was preserved in three cases [16, 18, 20]. Preservation of the mucosa, as in this report, should be ensured when possible to prevent the surgical field from becoming contaminated with intestinal contents, thus reducing the risk of infection and rectovaginal fistula. The mucosa is relatively easy to preserve by carefully peeling off the blunt or sharply detachable layers. However, 10–15% of GISTs reportedly show mucosal invasion and ulceration [2, 23], and mucosal preservation is not applicable in such cases. Instead of insisting on mucosal preservation, surgeons should consider tumor resection with negative margins to be of highest priority.

The following three points should also be noted when performing transvaginal tumor resection. First, the risk of bleeding is likely to be higher when resecting tumors that are closer to the lateral wall because this area contains more blood vessels than does the rectovaginal septum. The indications for the surgery should also be carefully considered because of the location of the tumor.

Additionally, we should also be aware of rectal stenosis associated with sutures. In this case, the rectal suture was performed while confirming the size of the rectal lumen by rectal examination. Although the rectal suture could not be closed horizontally because the lateral side was dissected only minimally as necessary, it was closed vertically after determining that the rectal lumen was sufficiently secured. If the risk of rectal stenosis is judged to be high, additional lateral dissection and horizontal suturing should be considered. Intraoperative endoscopy is also an option to check the rectal stenosis, although we did not perform since the rectum lumen could be adequately assessed by palpation. In this case, the later colonoscopy showed no stenosis.

Finally, the adverse effects on future pregnancy and childbirth should be considered when performing this procedure in young female. As shown in the table, there are no case reports on transvaginal resection of GIST in young women. A case report of transvaginal natural orifice translumenal endoscopic surgery, in which the posterior vaginal wall is incised similarly to transvaginal resection, described the risk of infertility is unknown, but avoidance of bleeding and inflammation to the pelvis should minimize this potential risk [24]. The report also mentioned the transvaginal approach is sometimes used for delivery of therapy to women with refractory infertility [25, 26], and transvaginal procurement of oocytes has been in practice for a long time [27], therefore based on these experiences in reproductive medicine, the risk of infertility from transvaginal manipulation seems to be very small. In this case, we consulted the gynecologist before the surgery and obtained consent for the procedure. Intraoperatively, the procedure was performed with the advice of the gynecologist, and postoperative management was also done in collaboration with the gynecologist.

## Conclusion

Transvaginal tumor resection is a treatment option as a minimally invasive procedure for GISTs in the anterior rectal wall in female patients.

## Data Availability

Not applicable (no datasets were generated during this study).

## Abbreviation

GIST: Gastrointestinal stromal tumor
